# ^1^H, ^13^C, ^15^N backbone resonance assignment of *apo* and ADP-ribose bound forms of the macro domain of Hepatitis E virus through solution NMR spectroscopy

**DOI:** 10.1007/s12104-022-10111-5

**Published:** 2022-10-22

**Authors:** Maria D. Politi, Angelo Gallo, Georgios Bouras, Maria Birkou, Bruno Canard, Bruno Coutard, Georgios A. Spyroulias

**Affiliations:** 1 Department of Pharmacy, University of Patras, 26504 Patras, Greece; 2 Department of Chemistry, University of Torino, 10126 Torino, Italy; 3 Université Aix-Marseille, Architecture et Fonction des Macromolécules Biologiques (AFMB) - UMR7257 CNRS - Case 932, 163 avenue de Luminy, 13288 Marseille CEDEX 09, France; 4 Unité des Virus Émergents (UVE: Aix-Marseille Univ-IRD 190-Inserm 1207), Marseille, France

**Keywords:** Macro domain, Hepatitis E virus, ADP-ribose, Solution NMR spectroscopy, Secondary structure

## Abstract

The genome of Hepatitis E virus (HEV) is 7.2 kilobases long and has three open reading frames. The largest one is ORF1, encoding a non-structural protein involved in the replication process, and whose processing is ill-defined. The ORF1 protein is a multi-modular protein which includes a macro domain (MD). MDs are evolutionarily conserved structures throughout all kingdoms of life. MDs participate in the recognition and removal of ADP-ribosylation, and specifically viral MDs have been identified as erasers of ADP-ribose moieties interpreting them as important players at escaping the early stages of host-immune response. A detailed structural analysis of the *apo* and bound to ADP-ribose state of the native HEV MD would provide the structural information to understand how HEV MD is implicated in virus-host interplay and how it interacts with its intracellular partner during viral replication. In the present study we present the high yield expression of the native macro domain of HEV and its analysis by solution NMR spectroscopy. The HEV MD is folded in solution and we present a nearly complete backbone and sidechains assignment for *apo* and bound states. In addition, a secondary structure prediction by TALOS + analysis was performed. The results indicated that HEV MD has a *α/β/α* topology very similar to that of most viral macro domains.

## Biological context

Hepatitis E virus (HEV) is the most common cause of acute viral hepatitis worldwide (Chandra et al. 2010). HEV is quasi-enveloped virus with a positive single-stranded RNA genome. It is the only member of the genus *Orthohepevirus* of the family *Hepeviridae* (LeDesma et al. 2019). According to World Health Organization (WHO), every year there are 20 million estimated cases of HEV infection, with 3.3 million symptomatic cases. The virus is transmitted via fecal–oral or zoonotic route. The latest is caused by close contact with infected animals or consumption of contaminated undercooked animal products (Doceul et al. 2016; Izopet et al. 2012; Yan et al. 2016). In general, HEV is self-limiting illness which lasts a few weeks. The incubation period is 2 to 6 weeks and the symptoms of hepatitis develop, with fever and nausea followed by abdominal pain, vomiting, anorexia, malaise, and hepatomegaly. About 40% of patients develop jaundice (Aslan and Balaban 2020). It is worth mentioning that there is a mortality excess in pregnant females and patients with chronic diseases (Chaudhry et al. 2015). In addition to the classical hepatic manifestations, HEV is responsible for extrahepatic disorders such as neurological disorders associated with Guillain—Barré syndrome and neuralgic amyotrophy (Narayanan et al. 2019; Sooryanarain and Meng 2019). No specific antiviral drug or vaccine is licensed globally for chronic hepatitis, underlining the necessity in the development of potent viral inhibitors.

The HEV genome is 7.2 kb long with a 7-methylguanosine cap at the 5′ end and is polyadenylated at the 3′ end. HEV consists of four open reading frames: ORF1, ORF2, ORF3 and ORF4. ORF4 is overlapped with ORF1 and its transcription is controlled by an IRES-like RNA structure with an essential role in HEV RNA polymerase proper function (Kenney and Meng 2019). ORF3 codes a 13 kDa small phosphoprotein, which enhances RIG-I signaling (VP13) (Nan et al. 2014a). ORF2 encodes a N-glycosylated 72 kDa protein important for the capsid formation, a protein that is an attractive target for HEV infection diagnostics and vaccine development (Nan and Zhang 2016). The larger ORF is the ORF1 that occupies about the 2/3 of the genome, encoding the non—structural protein crucial for viral replication, and composed of several functional domains. A methyltransferase (MeT/MTase), a Y undefined domain, a papain—like cysteine protease (PCP), a proline—rich hinge/hypervariable region (PPR/HVR), a macro domain, a helicase (Hel/NTPase) and an RNA-dependent RNA polymerase (Ojha and Lole 2016b; Wang and Meng 2021).

The HEV macro domain was identified as a putative interferon (INF) antagonist (Nan et al. 2014b). In addition, its C—terminal region displays direct interaction with both MTase and ORF3 proteins (Anang et al. 2016). HEV MD specifically interacts with the light chain subunit of human ferritin, and suppress its secretion in cultured cells (Ojha and Lole 2016a). HEV MD belongs to the ADP-ribose-1’’-monophosphatase (Appr-1''-pase family) that catalyses conversion of ADP-ribose-1′′-monophosphate (Appr-1′′-p) to ADP-ribose (Allen et al. 2003). Recent studies on protein ADP-ribosylation suggested that viral macro domains are able to de-ADP-ribosylate Asp or Glu side chain of host proteins, which brought them into focus as promising therapeutic targets (Fehr et al. 2018; Li et al. 2016).

In the last decade, the progress in the understanding of the crucial functions carried out by viral MDs, suggests that the MD could be a relevant antiviral target and stimulate the development of drug design efforts (Brosey et al. 2021; Dasovich et al. 2022; Fu et al. 2021; Ni et al. 2021; Rack et al. 2020).

Here, we present for the first time a ^1^H, ^13^C and ^15^N almost complete resonance assignment of the *apo* and ADP-ribose bound forms of HEV MD. These assignments should contribute to the understanding of the molecular mechanisms of de-ribosylation and provide starting points for inhibition or protein–protein interaction studies by NMR.

## Methods and experiments

### Protein expression and purification

The coding sequence of the HEV macro domain (HEV MD) (residues 772–926, Uniprot ID P29324) was synthesized, codon optimized (GenScript) and subcloned using *NdeI* and *XhoI* restriction enzymes into pET20b (+). The MD coding sequence is fused to an artificial ATG initiation codon in 5′ and to a sequence coding for an Hexahisitine preceded by a short linker (LE). Rosetta2 (DE3) (pLysS) *Escherichia coli* cells (Novagen) were transformed with the pET20b (+)—HEV MD. Precultures were grown overnight at 37 °C in 5 mL LB suppled with chloramphenicol and ampicillin, 180 revolutions per minute (rpm). Cells were then grown in 0.75 L minimal medium containing ^15^NH_4_Cl (1 g/L) and d-[^13^C_6_] glucose (4 g/L), NaCl (0.5 g/L), 1 mM MgSO_4_, 1.5 mL Solution Q [40 mM HCl, FeCl_2_·4H_2_O (50 mg/L), CaCl_2_·2H_2_O (184 mg/L), H_3_BO_3_ (64 mg/L), CoCl_2_·6H_2_O (18 mg/L), CuCl_2_·2H_2_O (4 mg/L), ZnCl_2_ (340 mg/L), Na_2_MoO_4_·2H_2_O (605 mg/L), MnCl2 4H_2_O (40 mg/L)] and was inoculated with the preculture and antibiotics (0.1 mg/mL ampicillin and chloramphenicol). Cell culture was grown at 37 °C, 200 rpm and when the Optical Density (OD) 600 reached 0.6–0.8, isopropyl β-d-1-thiogalactopyranoside (IPTG) was added to final concentration of 0.1 mM. After induction, the culture was incubated at 16 °C for seventeen hours (17 h). The cells were harvested by centrifugation at 8000 rpm for 10 min and pellet stored at − 80 °C until use.

Cell suspension was supplemented with 5% glycerol, 1 mM Tris (2-carboxyethyl) phosphine (TCEP) and EDTA-free protease cocktail (Sigma-Aldrich). Three freeze–thaw cycles (liquid N_2_ – 42 °C) were performed before the sonication step. Cells were then lysed by sonication and the cell debris was cleared by centrifugation (21.000 × *g*, 45 min, 4 °C). Supernatant was filtered through a 0.25 μm filter and loaded on a 5 mL His-Trap HF column (GE Healthcare) charged with Ni^2+^. The HEV MD was purified by immobilized metal affinity chromatography (IMAC) and eluted with 200 mM imidazole, 20 mM Na_2_PO_4_, pH 8.0, 500 mM NaCl, 1 mM TCEP, 1 mM phenylmethylsulfonyl fluoride (PMSF). The eluted HEV MD was gradually introduced to the NMR buffer (10 mM Sodium Acetate, 5 mM EDTA pH 5.4), using an Amicon Ultra 15 mL Centrifugal Filter membrane (Merck Millipore) and concentrated to a final volume of 1 mL. The protein was further purified by size exclusion chromatography using FPLC ÄKTA Purifier System (GE Healthcare) with Superdex® Increase 75 10/300 GL (GE Healthcare) pre-equilibrated with buffer 10 mM Sodium Acetate, 5 mM EDTA at pH 5.4. The protein was eluted according to its molecular weight, indicating a monomer. The fractions containing the HEV MD were collected and concentrated to a final volume of 500 μL and stored at − 80 °C. For the ADP-ribose bound state, a 100 mM stock solution of ADP-ribose sodium salt (Sigma A0752) was prepared in water. This stock solution was used to prepare the HEV MD—ADP-ribose complex by adding a tenfold molar excess to the protein.

### Data acquisition, processing and assignment

For the NMR experiments ^15^N and ^13^C/^15^N labelled samples prepared with a concentration of 0.4 mM for HEV MD in the *apo* form and 0.5 mM in the ADP-ribose bound form with protein to ADP-ribose ratio 1:10. All samples were in a mixed solvent of 90% H_2_O and 10% D_2_O (10 mM Sodium Acetate, 5 mM EDTA at pH 5.4). ^1^H chemical shifts were referenced on DSS methyl signal at 0.0 ppm. 0.25 mM 4,4-dimethyl-4-silapentane-1-sulfonic acid (DSS) were used as internal standard. ^13^C and ^15^ N chemical shifts were referenced indirectly to the ^1^H standard using a conversion factor derived from the ratio of NMR frequencies (Wishart et al. 1995). All NMR experiment were recorded on a Bruker Avance III HD 700 MHz NMR spectrometer equipped with a four-channel 5 mm cryogenically cooled TCI gradient probe at 298 Κ. All NMR data were processed with TOPSPIN 4.1.1 software and analysed with CARA 1.9.2a4 (Keller 2004). The acquired NMR experiments used for sequence specific assignment are summarized in Table 1. Backbone assignments and sidechains for HEV MD in the free and in the ADP-ribose bound form were obtained from the following series of heteronuclear experiments: 2D [^1^H,^15^N]–HSQC and 2D [^1^H,^15^N]–TROSY, 3D HN(CO)CA, 3D HNCA, 3D TROSY CBCA(CO)NH, 3D TROSY CBCANH, 3D HN(CA)CO, 3D HNCO, 3D HBHA(CO)NH, HCCH-TOCSY (Table 1).

**Table 1:** List of NMR experiments acquired at 700 MHz Bruker Magnet, including the main parameters used, to perform the sequence specific assignment of the backbone HEV MD in the free and ADPR bound forms

	Time domain data size (points)			Spectral width (ppm) and acquisition (ms)			ns	Delay time (s)	Experimental time
	t1	t2	t3	F1	F2	F3			
^1^H–^15^N HSQC	512	2048		44.0 (^15^ N) 90 ms	16.0 (^1^H) 104 ms		2	1.0	18’
^1^H–^15^N TROSY	256	1024		40.0 (^15^ N) 90 ms	14.0 (^1^H) 104 ms		4	1.0	36’
TROSY-HN(CO)CACB	96	40	1024	72.0 (^15^ N) 7 ms	42.0 (^15^ N) 4 ms	14.0 (^1^H) 52 ms	16	1.0	1d 14 h
TROSY-HNCACB	96	40	1024	72.0 (^15^ N) 7 ms	42.0 (^15^ N) 4 ms	14.0 (^1^H) 52 ms	16	1.0	1d 14 h
HN(CA)CO	64	40	1024	18.0 (^13^C) 10 ms	42.0 (^15^ N) 7 ms	14.0 (^1^H) 52 ms	16	1.0	1d 1 h
HNCO	64	40	1024	18.0 (^13^C) 10 ms	42.0 (^15^ N) 7 ms	14.0 (^1^H) 52 ms	8	1.0	12 h 30’
HNCA	80	40	1024	42.0 (^13^C) 5 ms	42.0 (^15^ N) 7 ms	14.0 (^1^H) 52 ms	8	1.0	7 h 49’
HN(CO)CA	80	40	1024	42.0 (^13^C) 5 ms	42.0 (^15^ N) 7 ms	14.0 (^1^H) 52 ms	16	1.0	15 h 52’
HBHA(CBCACO)NH	112	40	1024	8.0 (^1^H) 10 ms	42.0 (^15^ N) 7 ms	14.0 (^1^H) 52 ms	16	1.0	1d 21 h 33’
HCCH-TOCSY	128	48	1024	80.0 (^13^C) 5 ms	80.0 (^13^C) 2 ms	14.0 (^1^H) 52 ms	16	1.0	1d 5 h 32’

## Results

### Extent of assignments and data deposition

**Fig. 1:**
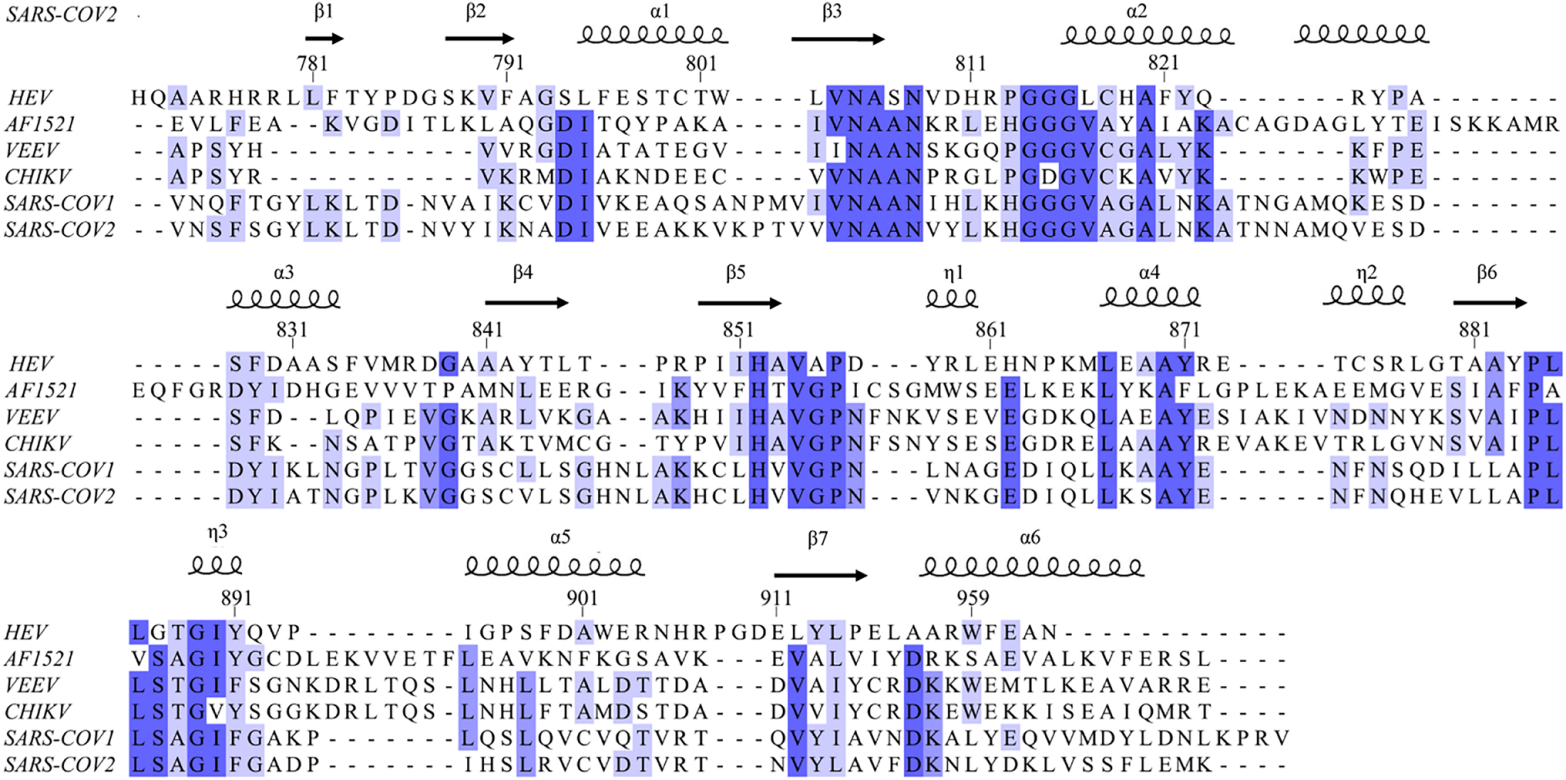
Sequence alignment of macro domains of Hepatitis E virus (HEV, Uniprot id: P29324), *Archaeoglobus fulgidus* (AF1521, Uniprot id: O28751), Venezuelan equine encephalitis virus (VEEV, Uniprot id: P36328), Chikungunya virus (CHIKV, Uniprot id: P36328) and the macro domain from the severe acute respiratory syndrome coronavirus (SARS-COV1/COV2 Uniprot id: P0C6X7/P0DTD1). All the alignment sequences are colored according to their identity percentages (> 80% mid blue, > 60% light blue, > 40% light grey and ≤ 40% white). The secondary-structure elements are labelled for SARS-CoV-2 (PDB entry: 6WEN)

The HEV macro domain shares a low sequence homology with other MDs (i.e., AF1521, VEEV, CHIKV, SARS-COV1, SARS-COV2) as shown in Fig. 1. Indeed, the percentage of identity between HEV MD and other viral MD is surprisingly low and found around 20% (23.44% with VEEV MD).

**Fig. 2:**
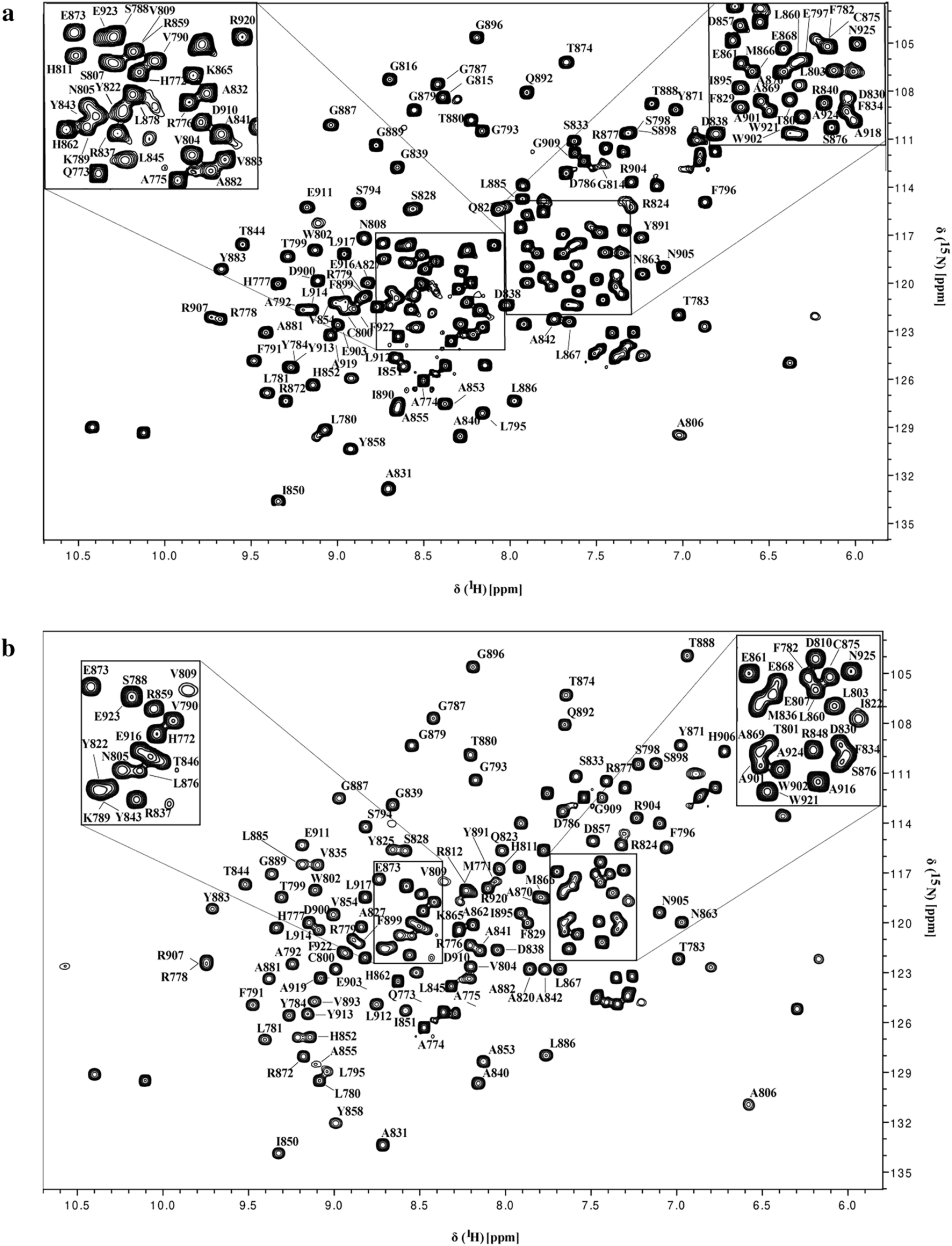
2D [^1^H-^15^N] HSQC spectrum at 298 K of HEV MD **a** *apo* form and **b** ADP-ribose bound form. *Left and right top* magnification of the central region of the 2D [^1^H-^15^N] HSQC spectrum

**Fig. 3:**
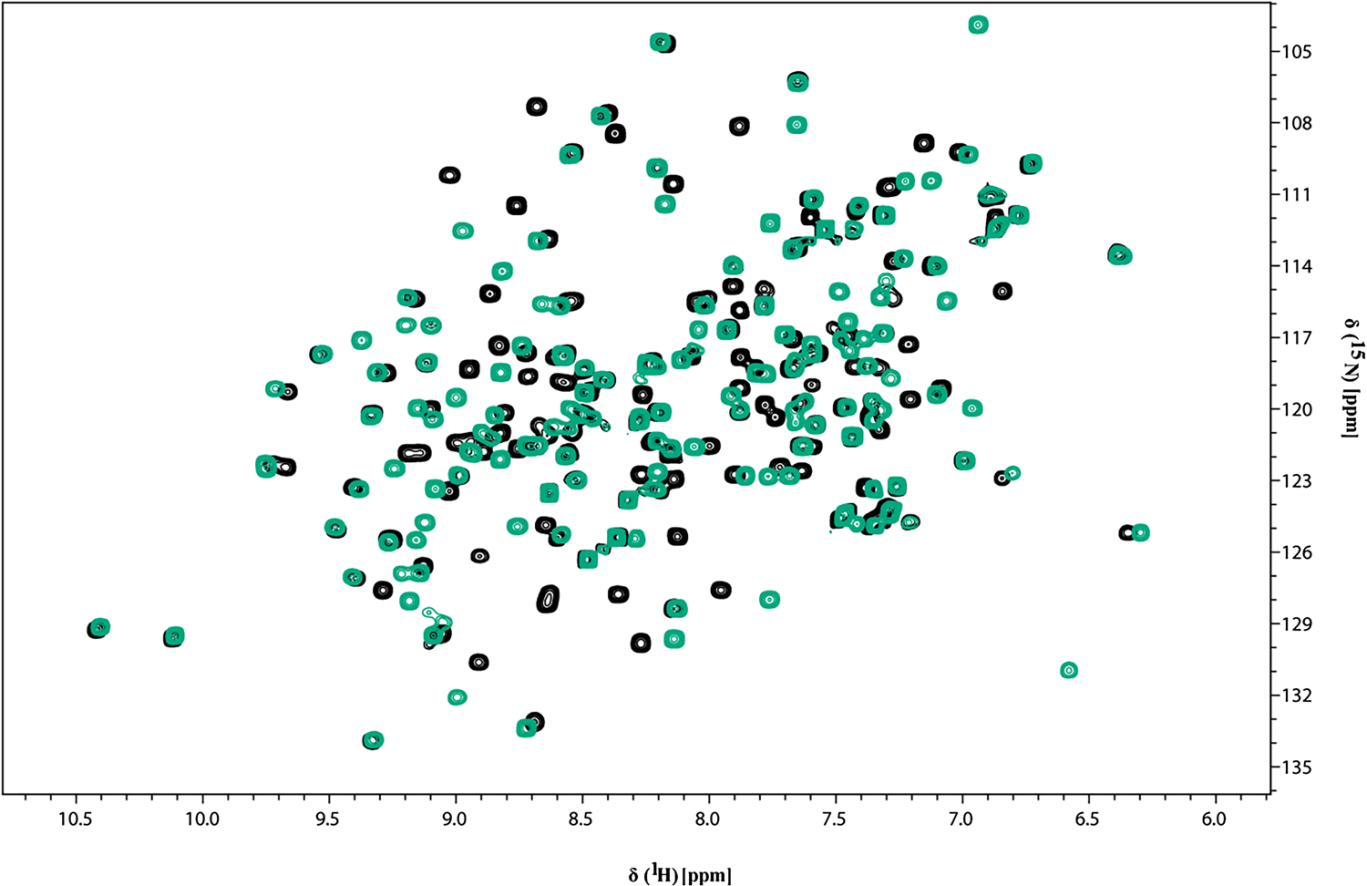
Superposition of the 2D [^1^H-^15^N] HSQC spectra of HEV MD in the absence (black) and presence of ADPR at a 1:10 molar ratio (green)

The NMR ^1^H–^15^N HSQC spectrum showed well-dispersed amide signals and narrows line widths, indicative of a well-folded monomeric polypeptide as shown in Fig. 2a for *apo* and in Fig. 2b for ADP-ribose bound form of HEV MD, respectively. In addition, the superposition of ^1^H–^15^N HSQC spectra of HEV MD in apo and bound state indicated significant chemical shift changes of the ^1^H–^15^N HSQC cross-peaks upon binding with ADPR, as shown in Fig. 3.

For the *apo* form of HEV MD, the analysis of the heteronuclear NMR experiments of the double isotopically labelled sample with the conventional backbone and sidechains methodology, results in the sequence specific assignment of 93.93% the resonances of the backbone atoms (HN, N, CO, Cα and Cβ) and 58.41% the resonances of the sidechains atoms. For the ADP-ribose bound form of HEV MD, we were able to assign 95.22% and 61.63% of the resonances of the backbone and sidechains atoms respectively.

The unassigned HN and N resonances of free HEV MD belong to D810, R812, L817, C818, H819, F821, T846. All the missing residues belong to loop regions or to unstructured regions or part of loops indicating some differences in their conformational dynamics features that hampers their detection. By contrary, the signals missing in the assignment of the ADP-ribose bound form of HEV MD belong to regions spanning only the residues S807, L817, C818, H819, F821. The disappearance of the above—mentioned set of resonances in the two forms might suggest conformational variability and flexibility upon binding.

**Fig. 4:**
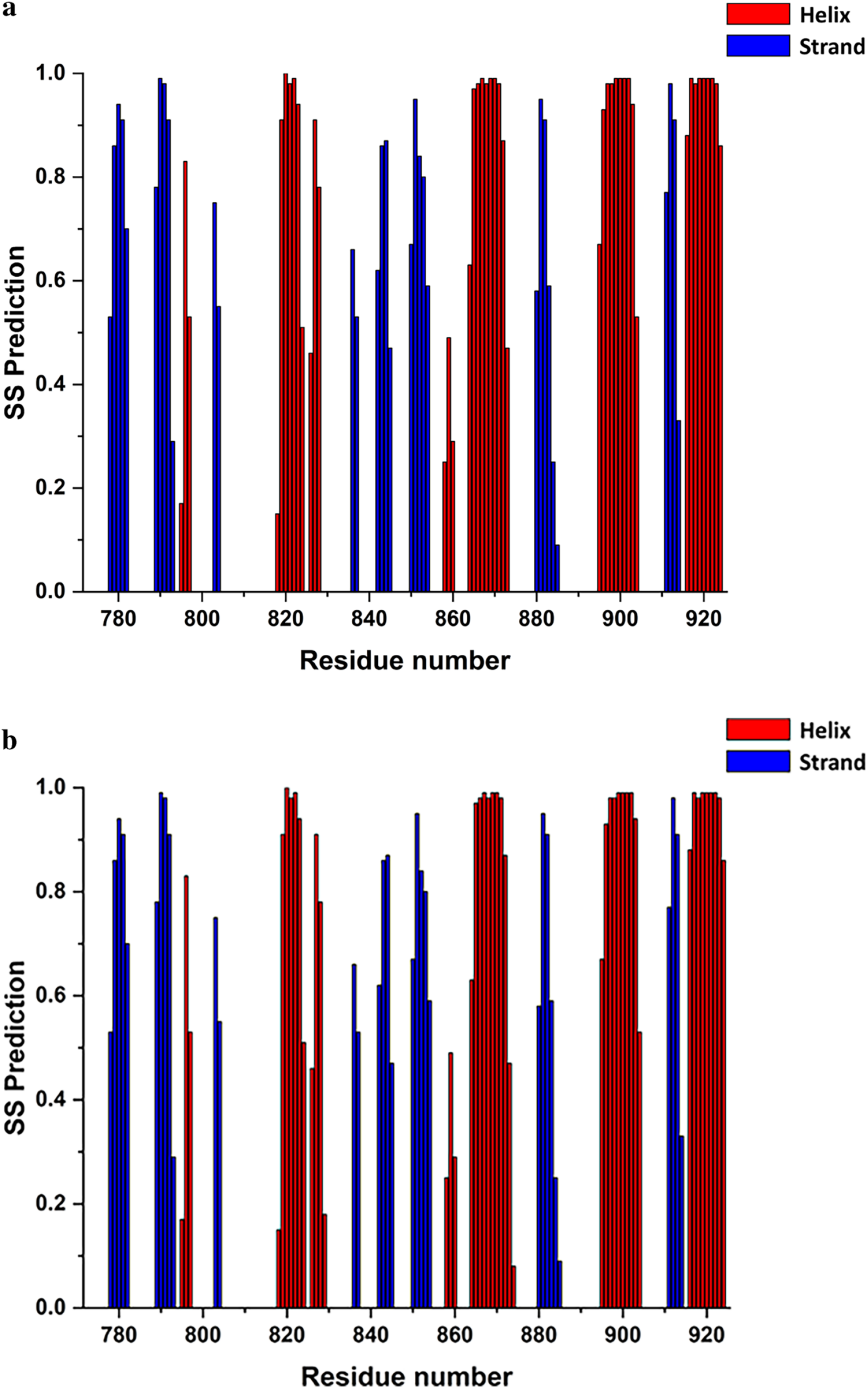
Predicted secondary structure of HEV MD using TALOS + . **a** *apo* form and **b** ADP-ribose bound form. Color coding red for *α*–helix and blue for *β–*sheets (red and blue bars indicate *α*-helix and *β-*strands, respectively)

In order to identify the secondary structure elements of the HEV MD *apo* and ADP-ribose forms, chemical shift assignments of backbone atoms (HN, Hα, Cα, Cβ, CO, N) for each residue in the sequence were analysed by TALOS + software (Shen et al. 2009). The secondary structure elements for free HEV MD protein are organized in an *α*/*β*/α sandwich-like fold with *β*/*β*/*α*/*β*/*α*/*β*/*β*/*β*/*α*/*β*/*α*/*β*/*α* topology from N- to C-terminal residues of the native sequence, graphically presented in Fig. 4. The order of the secondary structure segments are pretty similar to that of the other viral and human MDs ((Melekis et al. 2015), (Makrynitsa et al. 2015), (Lykouras et al. 2018), (Tsika et al. 2022)). We also report that upon interaction with ADPR no significant change in secondary structure elements has been identified (Fig. 3b). TALOS + analysis indicates also that HEV MD adopts a similar folding to that of many viral macro domains despite its low sequence similarity (Fig. 1), (Makrynitsa et al. 2019; Tsika et al. 2022).

Chemical shift values for the ^1^H, ^13^C and ^15^N resonances of HEV macro domain in the free state and in the ADPR bound state have been deposited at the BioMagResBank (https://www.bmrb.wisc.edu) under accession numbers 51470, and 51471, respectively.

To summarize, we present in this work a biological method to produce and purify in high yield the native form of recombinant HEV MD. NMR analysis indicated that the polypeptide is well folded and in monomeric state. These results will contribute to its 3D structure determination and open opportunities for the development of inhibitors with potential antiviral properties.

## Data Availability

Assignment deposited at the BioMagResBank under accession numbers 51470 and 51471.

## Abbreviations

HEV: Hepatitis E virus
MD: Macro domain
ADPR: Adenosine diphosphate ribose
ORF: Open reading frame
NMR: Nuclear magnetic resonance
OD: Optical density

## References

[CR1] Allen MD (2003). The crystal structure of AF1521 a protein from *Archaeoglobus fulgidus* with homology to the non-histone domain of MacroH2A. J Mol Biol.

[CR2] Anang S (2016). Identification of critical residues in hepatitis E virus macro domain involved in its interaction with viral methyltransferase and ORF3 proteins. Sci Rep.

[CR3] Aslan AT, Balaban HY (2020). Hepatitis E virus: epidemiology, diagnosis, clinical manifestations, and treatment. World J Gastroenterol.

[CR4] Brosey CA (2021). Targeting SARS-CoV-2 Nsp3 macrodomain structure with insights from human poly(ADP-ribose) glycohydrolase (PARG) structures with inhibitors. Prog Biophys Mol Biol.

[CR5] Chandra NS, Sharma A, Malhotra B, Rai RR (2010). Dynamics of HEV viremia, fecal shedding and its relationship with transaminases and antibody response in patients with sporadic acute hepatitis E. Virol J.

[CR6] Chaudhry SA, Verma N, Koren G (2015). Hepatitis E infection during pregnancy. Can Fam Phys Med Fam Can.

[CR7] Dasovich M (2022). High-throughput activity assay for screening inhibitors of the SARS-CoV-2 Mac1 macrodomain. ACS Chem Biol.

[CR8] Doceul V, Bagdassarian E, Demange A, Pavio N (2016). Zoonotic hepatitis E virus: classification, animal reservoirs and transmission routes. Viruses.

[CR9] Fehr AR, Jankevicius G, Ahel I, Perlman S (2018). Viral macrodomains: unique mediators of viral replication and pathogenesis. Trends Microbiol.

[CR10] Fu W (2021). The search for inhibitors of macrodomains for targeting the readers and erasers of mono-ADP-ribosylation. Drug Discov Today.

[CR11] Izopet J (2012). Hepatitis E virus strains in rabbits and evidence of a closely related strain in humans, France. Emerg Infect Dis.

[CR12] Keller, (2004). The computer aided resonance assignment tutorial.

[CR13] Kenney SP, Meng XJ (2019). Hepatitis E Virus genome structure and replication strategy. Cold Spring Harbor Perspect Med.

[CR14] LeDesma R, Nimgaonkar I, Ploss A (2019). Hepatitis E virus replication. Viruses.

[CR15] Li C (2016). Viral macro domains reverse protein ADP-ribosylation. J Virol.

[CR16] Lykouras MV (2018). NMR study of non-structural proteins-part III: (1)H, (13)C, (15)N backbone and side-chain resonance assignment of macro domain from Chikungunya virus (CHIKV). Biomol NMR Assign.

[CR17] Makrynitsa GI (2015). NMR study of non-structural proteins-part II: (1)H, (13)C, (15)N backbone and side-chain resonance assignment of macro domain from Venezuelan equine encephalitis virus (VEEV). Biomol NMR Assign.

[CR18] Makrynitsa GI (2019). Conformational plasticity of the VEEV macro domain is important for binding of ADP-ribose. J Struct Biol.

[CR19] Melekis E (2015). NMR study of non-structural proteins—Part I: 1H, 13C, 15N backbone and side-chain resonance assignment of macro domain from Mayaro virus (MAYV). Biomol NMR Assign.

[CR20] Nan Y, Ma Z (2014). Enhancement of Interferon Induction by ORF3 product of hepatitis E virus. J Virol.

[CR21] Nan Y, Ying Yu (2014). Hepatitis E virus inhibits type I interferon induction by ORF1 products. J Virol.

[CR22] Nan Y, Zhang Y-J (2016). Molecular biology and infection of hepatitis E virus. Front Microbiol.

[CR23] Narayanan S, Abutaleb A, Sherman KE, Kottilil S (2019). Clinical features and determinants of chronicity in hepatitis E virus infection. J Viral Hepatitis.

[CR24] Ni X (2021). Structural insights into plasticity and discovery of remdesivir metabolite GS-441524 binding in SARS-CoV-2 macrodomain. ACS Med Chem Lett.

[CR25] Ojha NK, Lole KS (2016). Hepatitis E Virus ORF1 encoded macro domain protein interacts with light chain subunit of human ferritin and inhibits its secretion. Mol Cell Biochem.

[CR26] Ojha NK, Lole KS (2016). Hepatitis E virus ORF1 encoded non structural protein-host protein interaction network. Virus Res.

[CR27] Rack JGM (2020). Viral macrodomains: a structural and evolutionary assessment of the pharmacological potential. Open Biol.

[CR28] Shen Y, Delaglio F, Cornilescu G, Bax Ad (2009). TALOS+: a hybrid method for predicting protein backbone torsion angles from NMR chemical shifts. J Biomol NMR.

[CR29] Sooryanarain H, Meng X-J (2019). Hepatitis E virus: reasons for emergence in humans. Curr Opin Virol.

[CR30] Tsika AC (2022). NMR study of macro domains (MDs) from betacoronavirus: backbone resonance assignments of SARS-CoV and MERS-CoV MDs in the free and the ADPr-bound state. Biomol NMR Assign.

[CR31] Wang Bo, Meng X-J (2021). Structural and molecular biology of hepatitis E virus. Comput Struct Biotechnol J.

[CR32] Wishart DS (1995). 1H, 13C and 15N random coil NMR chemical shifts of the common amino acids. I. Investigations of nearest-neighbor effects. J Biomol NMR.

[CR33] Yan B (2016). Hepatitis E virus in yellow cattle, Shandong, Eastern China. Emerg Infect Dis.

